# A Short Post-Reattachment Ultrasensitive Window of Time in Human Cancer Cells as Therapeutic Target of Prolonged Low-Dose Administration of Specific Compounds

**DOI:** 10.1155/2024/2699572

**Published:** 2024-02-05

**Authors:** Ashley Rebecca Guishard, Alecia Florence Guishard, Nina Semenova, Vivek Kaushik, Neelam Azad, Anand K. V. Iyer, Juan Sebastian Yakisich

**Affiliations:** ^1^School of Pharmacy, Hampton University, Hampton, VA, USA; ^2^Department of Pharmaceutical Sciences, School of Pharmacy, Hampton, VA, USA; ^3^Office of the Vice President for Research, Hampton University, Hampton, VA, USA

## Abstract

Prolonged low-dose administration (PLDA) of several FDA-approved drugs for noncancer conditions or dietary compounds is associated with a lower incidence of specific types of cancers and with the lower formation of metastasis. However, the underlying mechanism is unknown; there is a discrepancy between the concentration of drugs needed to kill cancer cells in vitro and the actual serum levels (10 and >1000 times lower) found in patients. In this study, we evaluated the hypothesis that clonogenicity may be the target of PLDA. We compared the effect of nigericin (NIG) and menadione (MEN) on the human A549 and H460 lung and MCF-7 and MDA-MB-231 breast cancer cell lines using routine MTT and colony forming assays (CFA). The ability of both NIG and MEN to eliminate 100% of cancer cells was at least 2-10 times more potent in CFA compared to MTT assays. Our results revealed the existence of a short post-reattachment window of time when cancer cells growing at low density are more sensitive to PLDA of specific drugs likely by targeting clonogenic rather than proliferation pathways. This short ultrasensitive window of time (SUSWoT) was cell- and drug-type specific: the SUSWoT for NIG was present in H460, A549, and MDA-MB-231 cells but not evident in MCF-7 cells. Conversely, a similar SUSWoT for MEN was present in MCF-7, MDA-MD-231, and A549 cells but not evident in H460 cells. Our findings partially explain the decreased incidence of specific types of cancer by PLDA of FDA-approved drugs (or dietary compounds) for noncancer conditions.

## 1. Introduction

Metastasis occurs through a sequence of events resulting in the development of an overt secondary tumor. As described by McGee et al. [1], cancer cells detach from the primary tumor, invade the adjacent tissue, and enter the lymphatic or circulatory system. Cells become arrested in distant organs due to size limitations. Here, they extravasate into the surrounding microenvironment, the conditions of which determine the extent to which the disseminated cells proliferate. Only one cell is necessary to proliferate into micrometastases (clinically undetectable) and potentially a secondary tumor, which may significantly impact cancer prognosis. We previously found that depending on the specific in vitro microenvironmental condition, cancer cells display different chemoresistance profiles. For instance, cells growing under adherent conditions are more sensitive compared to cells growing under suspension conditions [2–4]. On the other hand, cells growing in the absence of serum are more resistant compared to cells growing in the presence of serum [5, 6]. As metastasis is a multistep process, where cancer cells are at some point detach from the primary tumor, circulate in suspension, and reattach in a different tissue, it is expected that the chemosensitivity profile will vary depending on the step of the metastatic cascade. Thus, cancer cells will have specific short ultrasensitive windows of time (SUSWoT) where chemotherapy would be more effective. The identification of such SUSWoT will be of clinical importance: targeting this SUSWoT with specific drugs will improve the efficacy of anticancer drugs to prevent metastasis while decreasing their toxicity. In this article by using an *in vitro* model system that recapitulates the reattachment phase and the early step of the metastatic process, we identified a short post-reattachment window of time when cancer cells are more sensitive to specific anticancer drugs.

## 2. Materials and Methods

### 2.1. Cell Culture

The human NCI-H460 and A549 lung cancer cell lines as well as MDA-MB-231 and MCF7 breast cancer cell lines were purchased from American Type Culture Collection (Manassas, VA). RPMI 1640 and DMEM were purchased from Sigma-Aldrich (St. Louis, MO) and VWR (Radnor, PA). DPBS (Dulbecco's Phosphate Buffered Saline + calcium chloride and magnesium chloride) was purchased from Gibco. 100 mm culture dishes for adherent cell culture were purchased from Thermo Fisher Scientific.

### 2.2. Drugs

Menadione (2-methyl-1,4-naphthoquinone: vitamin K3) and nigericin were purchased from VWR (Radnor, PA) and stored as stock solution (25 and 100 mM, respectively) in DMSO at −20°C.

### 2.3. Cell Viability Assay: MTT

Dimethyl sulfoxide for molecular biology was purchased from Sigma-Aldrich. MTT (3-(4,5-dimethylthiazol-2-yl)-2,5-diphenyltetrazolium bromide) solution was also purchased from Sigma-Aldrich. 96-well culture plates were purchased from CELLTREAT Scientific Products (Pepperell, MA).

### 2.4. Colony Forming Assay

Crystal violet was purchased from Sigma-Aldrich. A crystal violet stock solution of 1% *w*/*v* in 20% ethanol in dH_2_O (100x) was prepared for the assay and was diluted to 0.1% *w*/*v* for colony staining. 37% formaldehyde was purchased from Ricca Chemical Company (Arlington, TX). A 3.7% formaldehyde (FA) solution in DPBS was used to fix the colonies. 6-well culture plates were purchased from VWR (Radnor, PA).

## 3. Methods

### 3.1. Cell Culture

NCI-H460 and A549 human lung cancer cells were cultured in RPMI 1640 supplemented with 5% and 10% fetal bovine serum (FBS), respectively. Culture media were supplemented with 2 mM L-glutamine, 100 U/mL penicillin, and 100 mg/mL streptomycin. MCF7 and MDA-MB-231 human breast cancer cells were cultured in 10% DMEM/high glucose supplemented with 10% FBS, 2 mM L-glutamine, 100 U/mL penicillin, and 100 mg/mL streptomycin. All cells were incubated in a 5% CO_2_ environment at 37°C. These supplemented culture media are referred to as “complete media” (CM).

### 3.2. MTT Assay

H460 and A549 (~2,000 cells/well) were plated in 96-well culture plates. MCF7 cells and MDA-MB-231 were plated at a density of 5,000 and 10,000 cells/well, respectively. After incubating the cells overnight in CM to allow them to adhere, the cells were treated with the appropriate concentrations of NIG or MEN and DMSO in sextuplicate wells. In all experiments, the highest concentration of DMSO was used as the control. Final drug dilutions were prepared in CM immediately before use. Cell viability was evaluated using the MTT assay. After 72 hours, the media in each microwell was replaced with 100 *μ*L of a 1 : 5 dilution of MTT stock solution in CM. After incubation at 37°C and 5% CO_2_ for ~1 hour, the MTT solution was replaced with 100 *μ*L DMSO and incubated at 37°C and 5% CO_2_ for 10 minutes. The absorbance of the solubilized formazan was then read at 570 nm (reference wavelength 690 nm) using an ELISA (enzyme-linked immunosorbent assay) multiwell plate reader (Synergy-1; BioTek). All experiments were performed at least three times.

### 3.3. Colony Forming Assay

The colony forming assay was performed according to Rafehi et al. [7]. Approximately 50 cells/well of H460 cells, 500 cells/well of MDA-MB-231, and 200 cells/well of A549 and MCF7 cells were plated in 6-well culture plates and allowed to adhere overnight. Then, they were treated with the appropriate concentrations of NIG or MEN and DMSO for 72 hours. Final drug dilutions were prepared in CM immediately before use. The control experiments contained the highest concentration of DMSO only. After treatment, the media in one-third of the 6-well plates were replaced with drug-free CM (DFM), and the colonies were allowed to grow for ~7-11 more days (cell line dependent). DFM was replaced twice per week (approximately every 72 hours). After a total of 10 days for H460 and A549 or 14 days for MCF7, all media were removed. The colonies were washed twice with DPBS and fixed with 3.7% formaldehyde for 1 hour at room temperature and then stained with 0.01% crystal violet for 1 hour. The plates were allowed to dry overnight, and then, the colonies were photographed and quantified with the current version of ImageJ software (Fiji ImageJ, version 1.53o). MDA-MB-231 cells did not form visible colonies after 10 days of treatment. Thus, regrowth experiments in drug-free media were conducted (see Results and Discussion).

**Figure 1:**
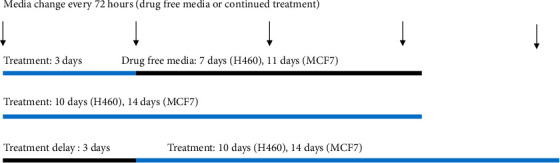
Schematic of CFA experimental design for H460 and MCF7 cells. Cells were treated for 3 days, or for 10 days with or without a 3-day delay. The arrows indicate medium changes with fresh drugs every 72 hours. A similar design was used for two additional cell lines (A549 and MDA-MB-231 cells).

To determine the effect of prolonged treatment on clonogenicity, one-third of the 6-well plates underwent continued treatment (CT), whereby, after 72 hours, the media were replaced with the same treatment regimens of NIG or MEN and DMSO. Drug-containing media were replaced twice per week for ~7-11 additional days. After 10 days for H460, A549, and MDA-MB-231 and 14 days for MCF7, all media were removed and the cells/colonies were fixed, stained, and counted. To determine the effect of delaying treatment of plated cells on clonogenicity, cells in the final third of 6-well plates were allowed to incubate for 72 hours before being treated with the same treatment regimen of NIG or MEN and DMSO. These plates underwent CT as well, with drug-containing media replaced twice per week for a total of 10 days (H460, A549, and MDA-MB-231) or 14 days (MCF7) of treatment, after which the colonies were fixed, stained, and quantified.  Figure 1 summarizes the experimental setup to study prolonged treatment with and without delay. In all experiments, the highest concentration of DMSO was used as the control. All experiments were performed at least three times.  Figure 1 displays a schematic representation of the experimental design for the colony forming assays.

### 3.4. Colony Counting and Digital Imaging

Digital images of the colonies were captured using a camera and counted using the imaging analysis software ImageJ (Fiji, version 1.53o) as previously described [8].

## 4. Results and Discussion

Our in vitro experimental design using CFA in combination with PLDA of anticancer compound partially mimics the late phase of metastasis formation occurring *in vivo* when a single cancer cell reattaches and starts proliferating in a new distant organ. While the MTT assay measures cell viability and proliferation [9], the CFA is a clonogenic assay that measures the ability of a single cell to produce a colony [10]. Treatment of cells in CFA experiments with PLDA drugs partially mimics the clinical situation of patients receiving long-life administration of drugs for noncancer conditions. This strategy allowed us to study in vitro the effect of PLDA of specific drugs on clonogenicity. For technical limitations, such studies would be difficult to perform *in vivo* due to the lack of techniques with ability to identify and follow the fate of a single cell implanted in a new organ.

### 4.1. Effect of *NIG* on H460 Lung Cancer Cells

**Figure 2:**
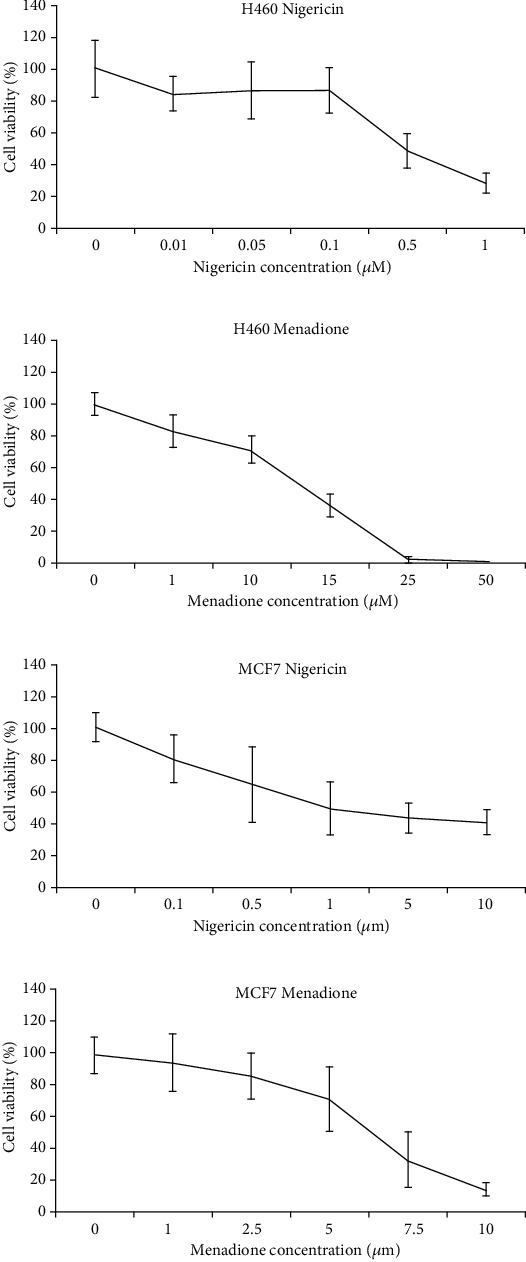
MTT assay. After incubating ~2,000 cells/well in 96-well culture plates overnight to adhere, H460 cells were exposed to increasing concentrations of (a) nigericin and (b) menadione. For MCF7, ~5,000 cells/well were plated in 96 well plates, allowed to adhere, and then exposed to (c) nigericin and (d) menadione. After 72 hours, the MTT assay was performed. This figure is representative of at least three independent experiments.

The antiproliferative effects of NIG on H460 lung cancer cells were evaluated using the MTT assay.  Figure 2(a) is a representative selection from six independent assays. Treatment with 0.5 *μ*M NIG and 1 *μ*M NIG for 72 hours decreased the viability of H460 by approximately 55% and 71% on average, respectively. Approximately 20-30% of viable cells were present even at the highest concentration tested (1 *μ*M).

**Figure 3:**
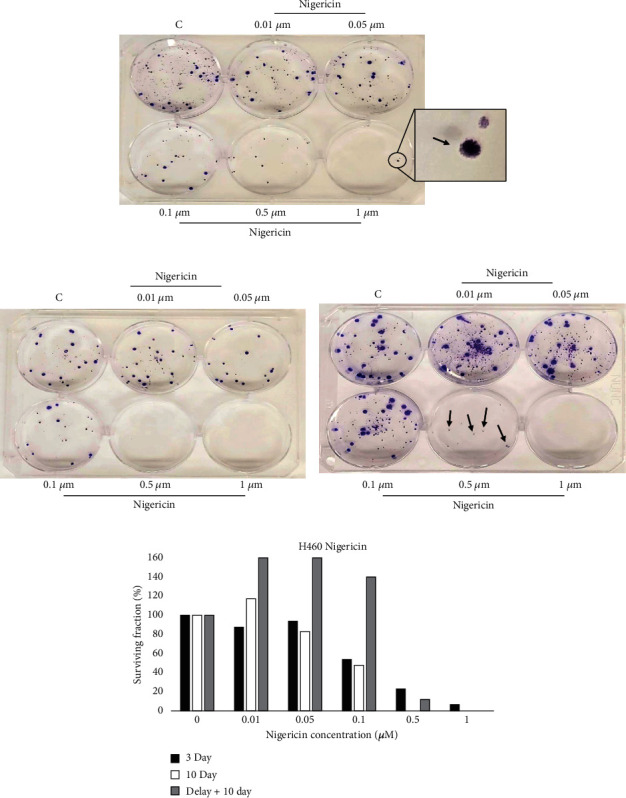
Existence of a SUSWoT in H460 cells treated with nigericin. Fifty cells were plated per well and allowed to adhere overnight. Then, cells were exposed to the indicated concentrations of *nigericin* for 3 days followed by incubation in drug-free media for (a) 7 days, (b) 10 days, or (c) 10 days following a 3-day period of drug-free medium incubation. Afterwards, colonies were stained with crystal violet and quantified with ImageJ software (d). This figure is representative of three independent experiments.

The anticlonogenic activity of NIG on H460 cells was assessed using the CFA.  Figure 3 is a representative visual selection from three independent experiments. In CFA, on average, one colony was detected in H460 cells exposed to 1 *μ*M for 3 days (see arrow in Figure 3(a)). Treatment of H460 cells with 1 *μ*M NIG for 10 days with or without delay completely prevented colony formation (Figures 3(b) and 3(c)). On average, exposure of H460 cells to 0.5 *μ*M NIG for 3 days decreased the formation of colonies by approximately 72%. Colonies were not observed when H460 cells were exposed to 0.5 *μ*M NIG for 10 days. This result may be expected because the longer the cells are exposed to the drugs, more cells would die and less colonies would develop. However, when H460 cells were exposed for 10 days with 0.5 *μ*m NIG but after 3 days of plating (prolonged treatment with delay), approximately 3-4 colonies on average were observed. Additionally, larger colonies were present in H460 cells treated with NIG following delayed 10-day treatment compared to both 3-day treatment and 10-day treatment without delay (Figure 3(c)).

Compared to the MTT assay, the ability of NIG to eliminate H460 cancer cells was more potent in CFA on average. Exposure of H460 cells to NIG at 1 *μ*M was insufficient to eliminate 100% of cells in the 72-hour MTT assay (5,000 cells/well density), while 10-day treatment with or without delay at this concentration in the CFA was. Yakisich et al. [8] previously described the ability of NIG to reduce the viability of H460 cells in a concentration-dependent manner. However, exposure of H460 cells to 50 *μ*M of NIG was not sufficient to eliminate 100% of cells (Figure 1(a)) in Yakisich et al. [8].

In this experiment, the results indicate that to prevent H460 colony formation, treatment with NIG should be initiated within a 3-day time window and must continue at a concentration of at least 0.5 *μ*M for at least 10 days to be most effective. Initiating treatment at appropriate concentrations within this period is critical for preventing colony formation since delaying treatment for just 3 days allows for colony formation at otherwise effective concentrations. The presence of larger colonies following delayed 10-day exposure compared to 10 days without delay implies that low cell density (50 cells/well) alone is not a determinant for the greater potency of H460 cancer cell elimination in the CFA relative to the MTT assay. We conclude that a short (3 days) ultrasensitive window of time (SUSWoT) wherein H460 cells are more sensitive to the PLDA of NIG exists in cancer cells. To further investigate whether the SUSWoT is present in another cell line and/or restricted to specific drugs such as *NIG*, we performed additional experiments using another drug (MEN) as well as another cell line (breast cancer cell line MCF-7).

### 4.2. Effect of MEN on H460 Lung Cancer Cells

**Figure 4:**
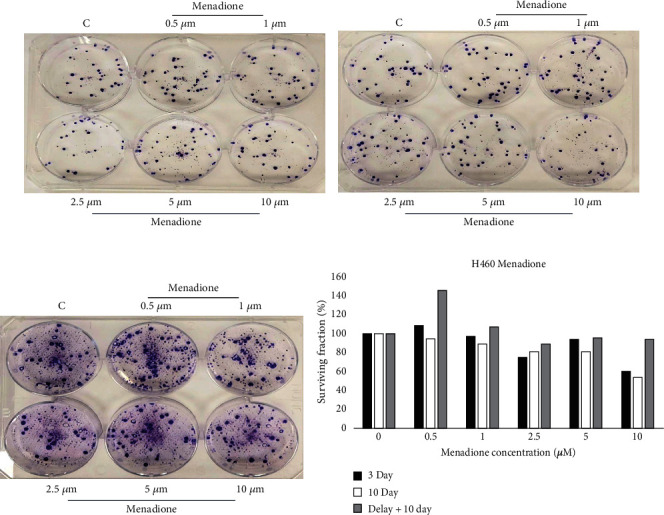
Absence of a SUSWoT in H460 cells treated with menadione. Cells were exposed to the indicated concentrations of menadione, stained, and quantified in a similar fashion as Figures 3(a)–3(d). This figure is representative of three independent experiments.

Next, to evaluate whether other drugs may show a similar profile in H460 lung cancer cells, we evaluated the effect of MEN using the same experimental approach: MTT assay was used to assess the effect of MEN on cell viability in H460 cells.  Figure 2(b) is a representative figure from three independent 72-hour MTT assays. On average, treatment of H460 cells with 1 *μ*M, 10 *μ*M, and 15 *μ*M of MEN decreased cell viability by approximately 12%, 32%, and 59%, respectively. The anticlonogenic activity of MEN on H460 cells was assessed using the colony forming assay.  Figure 4 is a representative visual selection from three independent experiments. Exposure of H460 cells to 1 *μ*M and 10 *μ*M MEN for 3 days decreased average colony formation by approximately 1% and 47%, respectively (Figure 4(a)). Treatment of H460 cells with 10 *μ*M MEN for 10 days *with* delay (Figure 4(c)) produced a similar average reduction in colony formation of 45% (7% colony reduction relative to control in the representative Figure 4(d)), but cell treatment with 1 *μ*M reduced colony formation by approximately 20% (7% more colonies than control in the representative (Figure 4(d))). On average, 10-day exposure of H460 cells to 1 *μ*M and 10 *μ*M MEN without delay decreased colony formation by approximately 8% and 56%, respectively (Figure 4(b)). Across all CFA replicates, treatment of H460 with MEN failed to produce a consistent, concentration-dependent reduction in colony formation.

On average, the ability of MEN to eliminate H460 cancer cells was almost equally as potent in CFA compared to MTT. When treated with 1 *μ*M MEN in MTT, approximately 88% of viable cells were present after 72 hours. In CFA, approximately 99% of viable cells were present on average after exposure to 1 *μ*M MEN for 3 days. Approximately 69% of viable cells were present when H460 cells were exposed to 10 *μ*M MEN in the MTT assay compared to 53% of viable cells remaining in CFA with 3-day treatment. In both CFA and MTT assays, exposing cells to the highest concentrations of MEN tested (50 *μ*M for MTT, 10 *μ*M for CFA) was insufficient to eliminate 100% of cells across all experiments.

There was no considerable difference in the surviving fraction of colonies when short-term treatment with MEN (3 days) was used compared to prolonged treatment (10 days). Additionally, both the data and visual inspection of the colonies suggest that delaying treatment for 3 days (followed by a treatment period of 10 days) appeared to promote resistance to MEN. In contrast with the treatment of H460 cells with NIG, there was no 3-day window of time where H460 exhibits heightened sensitivity to MEN. These results demonstrated that the SUSWoT is drug-type dependent and suggest that it may also be cell-type dependent. In order to test this ad hoc hypothesis, we next tested the effect of MEN and NIG on MCF-7 breast cancer cells.

### 4.3. Effect of MEN on MCF7 Breast Cancer Cells

**Figure 5:**
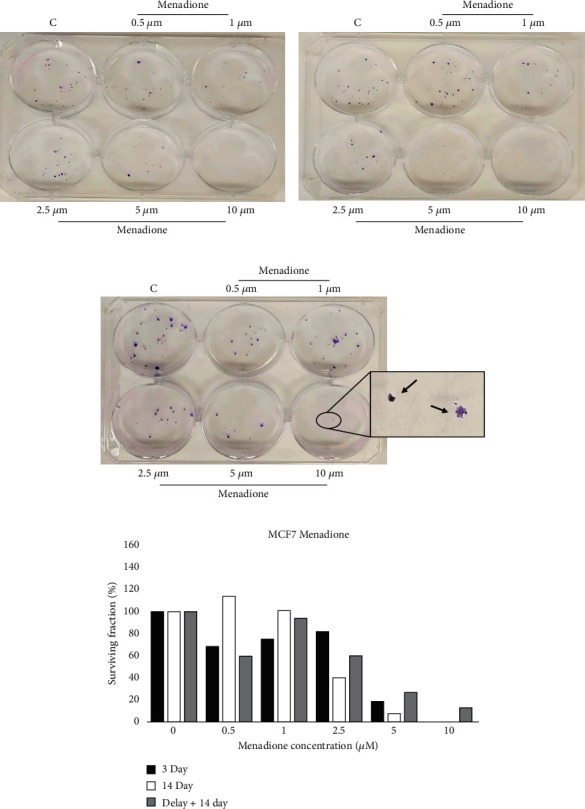
Existence of a SUSWoT in MCF7 cells treated with menadione. Cells were exposed to the indicated concentrations of menadione, stained, and quantified in a similar fashion as Figures 3(a)–3(d). This figure is representative of three independent experiments.

Figure 2(d) represents the average of three independent MTT assays evaluating the cell viability of MCF7 after treatment with MEN. Exposure to 5 *μ*M MEN produced an average reduction in cell viability of approximately 29%, while treatment with 7.5 *μ*M MEN decreased the viability of MCF7 by approximately 68%. The anticlonogenic activity of MEN on MCF7 cells was assessed using the colony forming assay.  Figure 5 is a representative visual selection chosen from three independent CFA assays. In CFA, MEN at 10 *μ*M for 3 days and 14 days without delay could completely prevent colony formation (Figures 5(a) and 5(b)). However, on average, 3 (2-4) colonies were present when MCF7 cells were treated with 10 *μ*M MEN for 14 days following a 3-day delay (Figure 5(c)). Exposure of MCF7 to 5 *μ*M MEN for 3 days, 14 days *with* 3-day delayed initiation, and 14 days *without* delay decreased colony formation by approximately 60%, 76%, and 87%, respectively. Additionally, the colonies in the 3-day delay plates across all replicates were more considerable visually than those in the 3-day and 14-day treatment plates.

The ability of MEN to eliminate MCF7 cancer cells was approximately 2-14 times more potent in CFA than in MTT assay on average. In the MTT assay, the highest concentration of MEN tested (10 *μ*M) was insufficient to eliminate all cells (13.9% viable cells remained on average). Approximately 77% of viable cells remained when MCF7 was treated with 5 *μ*M MEN in 72-hour MTT compared to 40% viable colonies in 3-day CFA.

There was no apparent difference in cell survival when treating MCF7 cells with MEN for 3 or 14 days, especially at higher MEN concentrations. This lack of difference suggests that continuous treatment with MEN is not necessary to kill cells. A potential explanation for this observation is that short-term exposure to MEN produced irreversible damage. This interpretation is in agreement with our previous finding that removal of MEN after a short incubation time failed the ability to synthesize DNA in rat cerebral cortex tissue [11]. However, if 14-day treatment is started after the 3-day time window, MCF7 exhibits some chemoresistance to MEN. We conclude that there is a 3-day time window (a SUSWoT) where MCF7 cells exhibit increased sensitivity to MEN exposure.

### 4.4. Effect of NIG on MCF7 Breast Cancer Cells

**Figure 6:**
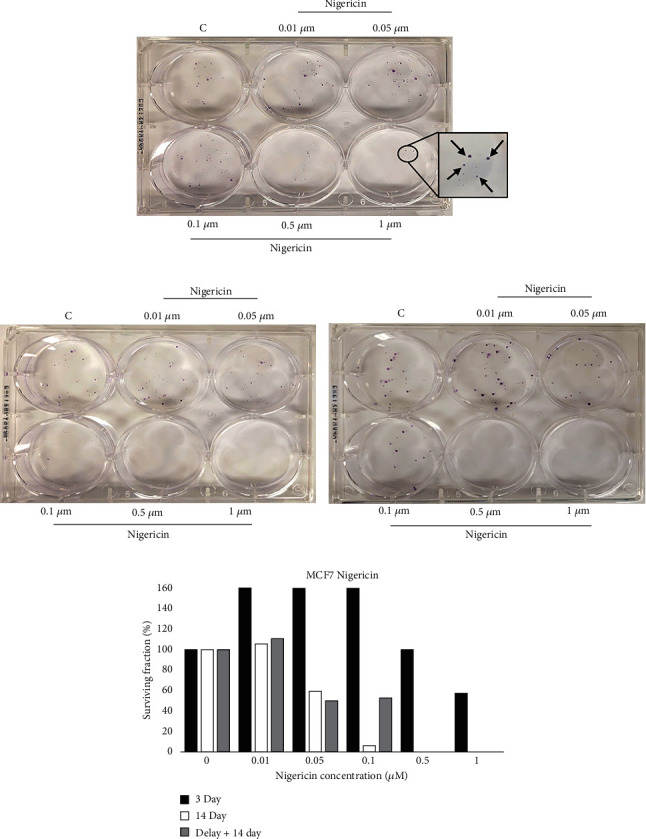
Absence of SUSWoT in MCF7 cells treated with *nigericin*. Cells were exposed to the indicated concentrations of nigericin, stained, and quantified in a similar fashion as Figures 3(a)–3(d). This figure is representative of three independent experiments.

The effect of NIG on MCF7 breast cancer cell viability and proliferation was evaluated using the MTT assay.  Figure 2(c) is the average of three independent experiments. The average cell viability of MCF7 decreased by approximately 50% when treated with 1 *μ*M NIG for 72 hours. Exposure to 10 *μ*M NIG, the highest concentration evaluated, was insufficient to eliminate 100% of MCF7 cells in the MTT assay. At this concentration, MCF7 cell viability decreased by 59% on average. The anticlonogenic activity of NIG on MCF7 cells was assessed using the CFA. The plates selected for Figure 6 visually represent three independent CFA assays. Exposure of MCF7 cells to 0.5 *μ*M and 1 *μ*M for 14 days with or without delay was sufficient to prevent colony formation entirely (Figures 6(b) and 6(c)). On average, approximately 18 and 7 colonies were present when MCF7 cells were treated with 0.5 *μ*M and 1 *μ*M NIG (respectively) for 3 days, followed by drug-free media for 11 days (Figure 6(a)).

Interestingly, the ability of NIG to eliminate MCF7 cancer cells on average was ~1.5 times more potent in the MTT assay compared to the CFA. After exposure to 0.1 *μ*M NIG for 72 hours in the MTT assay, approximately 81% of viable cells remained, compared to a 21% *increase* in colony formation exceeding the control on average in the 3-day CFA. Similarly, exposure to 0.5 *μ*M NIG in the MTT assay reduced cell viability by approximately 35%, while the same concentration in the CFA decreased colony formation by only 4% on average.

Prolonged treatment for 14 days with NIG is necessary to prevent colony formation of MCF7 cells. Treatment with NIG for only 3 days at any evaluated concentration was insufficient to eliminate colony formation. Delaying treatment for 3 days prior to initiating a 14-day treatment regimen had little impact on colony formation and survival compared to 14 days of treatment without delay, indicating that no time window exists where MCF7 exhibits chemosensitivity to NIG. In summary, the results from the above experiments demonstrated that H460 lung cancer cells display a SUSWoT for NIG but not for MEN and MCF-7 breast and SUSWoT for MEN but not for NIG. Thus, the SUSWoT is cell-specific as well as drug-specific.

### 4.5. The Existence of the SUSWoT in Additional Cell Lines

The existence of the SUSWoT was investigated in two additional human cancer cell lines (A549 lung and MDA-MB-231 breast cancer) that were treated with MEN and NIG using the same experimental approach. The short-term antiproliferative effects of MEN and NIG were evaluated by the MTT assay (Figure S1) and then by the CFA (Figure S2-S6). Once again, a delayed treatment for 3 days prior to initiating a prolonged treatment (7-10 days) revealed the existence of a SUSWoT in both cell lines. A SUSWoT was observed in A549 cells treated with either NIG or MEN (Figure S2-S3).

MDA-MB-231 cells did not form visible colonies by the end of the treatment. However, because viable cells were observed under microscopic examination (Figure S4), all plates were incubated in drug-free media for an additional 10 days. These “regrowth experiments” revealed the existence of a SUSWoT when MDA-MB-231 cells were exposed to both NIG and MEN (Figure S5-S6).

### 4.6. Drug and Cell Line Specificity: Clinical Implications

From the clinical point of view, our results agree with extensive epidemiological data that showed that specific FDA-approved drugs, when prescribed for noncancer conditions, only decrease the incidence of few types of cancer but have no effect of other cancer types. For instance, metformin was found to decrease the incidence of prostate, colorectal, and breast cancers [12–14] but not of others. A similar profile was observed with many other FDA-approved drugs (reviewed by Chang et al. (2022), unpublished data). Taken together, our results reinforce the conclusion that no single chemoprophylactic agent can prevent colony formation in all types of cancer. However, our data showed that it would be possible to identify specific drugs that may prevent *in vitro* the formation of colonies. These specific drugs can be exploited in the clinic to decrease the incidence of metastasis in specific cancers. The clinical efficacy of tamoxifen after surgery and radiation to reduce the risk of invasive breast cancer supports this conclusion [15, 16]. In women with breast cancer, the mean concentrations of tamoxifen in plasma and venous blood were 89.31 ng/mL and 98.31 ng/mL, respectively [17]. After 28 days of treatment with a dose of 20 mg/day, the serum concentration was 83.6 (8.7–134.4) ng/mL [18]. However, in vitro, the concentration needed to decrease the viability of MCF-7 and MD-231 breast cancer cells after 72 h of treatment was higher than 500 ng/mL [19]. These data from the literature suggest that tamoxifen is between 3 and 11 times more potent in patients (likely due to the long-term exposure) compared to its *in vitro* activity (measured after 72 h).

In this regard, our data suggest that NIG may be an effective drug for preventing metastasis in H460 lung cancer and that MEN may be useful in the prophylaxis of MCF7 breast cancer metastases. While NIG is an experimental drug that may require further investigation before approved for clinical use, MEN has been tested in combination with vitamin C (Apatone®) in clinical trials with relative safety. Oral administration of Apatone showed promise in treating prostate and advanced inoperable bladder cancers [20]. In addition, other drugs that received FDA approval for noncancer condition can be safely administered for prolonged period of time (some of them long life, e.g., metformin and aspirin). This fact points to the necessity of initiating therapy within the identified SUSWoT, as cancer cells, including H460 lung cancer and MCF7 breast cancer, begin forming colonies and likely displaying heterogeneity just three days after single-cell adhesion.

Clinically, PLDA with specific therapies is essential to preventing tumor progression, as discontinuation of specific therapies may lead to cancer recurrence and metastatic potential. For example, in the global Adjuvant Tamoxifen: Longer Against Shorter (ATLAS) trial, it was determined that continuing tamoxifen therapy for 10 years instead of 5 years reduced the risk of cancer recurrence, reduced breast cancer mortality, and reduced overall mortality of women with ER-positive breast cancer after year ten [21].

## 5. Conclusions

Our data demonstrated that a SUSWoT exists immediately after cancer cells resume anchorage-dependent culture conditions. This SUSWoT is cell type and drug specific and can potentially be exploited to prevent the risk of invasive cancer after surgery and routine radio and chemotherapy cycles. Our findings partially explain the decreased incidence of specific types of cancer in patients taking lifelong therapy with FDA-approved drugs (or dietary compounds) for noncancer conditions: the continuous presence of the drug at low concentrations eliminates cells during the SUSWoT, when cancer cells are highly sensitive. We propose that PLDA of drugs with specific compounds targeting clonogenicity of specific cancer cell types may be a useful and safe clinical strategy to reduce the mortality of cancer patients by decreasing or preventing metastasis.

## Data Availability

The data that support the findings of this study are available from the corresponding author upon reasonable request.

## Supplementary Materials

Supplementary Materials Figure S1-S6: existence of a SUSWoT in A549 lung and MDA-MB-231 breast cancer cell lines.
