# Supplementary material for: A Short Post-Reattachment Ultrasensitive Window of Time in Human Cancer Cells as Therapeutic Target of Prolonged Low-Dose Administration of Specific Compounds
Source: Int J Cell Biol. 2024 Feb 5;2024:2699572. doi: 10.1155/2024/2699572 (PMC10861276; doi:10.1155/2024/2699572)
Supplement: Supplementary Materials — Figure S1-S6: existence of a SUSWoT in A549 lung and MDA-MB-231 breast cancer cell lines. [file 2699572.f1.docx]

**Supplementary Material**: Existence of a SUSWoT in A549 lung and MDA-MB-231 breast cancer cell lines.

**Figure S1.** MTT Assay. After incubating ~2,000 cells/well in 96-well culture plates overnight to adhere, A549 cells were exposed to increasing concentrations of (a) Menadione (b) Nigericin. For MDA-MB-231, ~10,000 cells/well were plated in 96 well plates, allowed to adhere, and then exposed to (c) Menadione and (d) Nigericin. After 72 hours, the MTT assay was performed. This figure is representative of at least three independent experiments.

**Figure S2.** *Existence of a SUSWoT in A549 cells treated with Nigericin*. Two hundred cells were plated per well and allowed to adhere overnight. Cells in plate (a) were treated for three days followed by incubation in drug free media. Cells in plates (b) and (c) were treated for prolonged periods without or with an initial period of three days in drug-free media, respectively. Afterwards, colonies were stained with crystal violet and quantified with ImageJ software (d). This figure is representative of four independent experiments.

**Figure S3.** *Existence of a SUSWoT in A549 cells treated with Menadione*. Two hundred cells were plated per well and allowed to adhere overnight. Cells in plate (a) were treated for three days followed by incubation in drug free media. Cells in plates (b) and (c) were treated for prolonged periods without or with an initial period of three days in drug-free media, respectively. Afterwards, colonies were stained with crystal violet and quantified with ImageJ software (d). This figure is representative of four independent experiments.

**Figure S4. MDA-MB-231 cells do not form visible colonies.** Microscopic images of untreated cells (20 x). Each image represents a random field taken from different plates day 11 after plating 500 cell/well in 6-well plates.

**Figure S5.** *Existence of a SUSWoT in MDA-MD-231 cells treated with Nigericin*. Five hundred cells were plated per well and allowed to adhere overnight. Cells in plate (a) were treated for three days followed by incubation in drug free media. Cells in plates (b) and (c) were treated for prolonged periods without or with an initial period of three days in drug-free media, respectively. After the treatment, all cells were incubated in drug-free media for 10 days (Regrowth phase). Afterwards, cells were stained with crystal violet. The visible dots resembling colonies are overgrown areas of cells but not real colonies because they did not originate from a single cell. This figure is representative of four independent experiments.

**Figure S6.** *Existence of a SUSWoT in MDA-MD-231 cells treated with Menadione*. Five hundred cells were plated per well and allowed to adhere overnight. Cells in plate (a) were treated for three days followed by incubation in drug free media. Cells in plates (b) and (c) were treated for prolonged periods without or with an initial period of three days in drug-free media, respectively. After the treatment, all cells were incubated in drug-free media for 10 days (Regrowth phase). Afterwards, the colonies were stained with crystal violet. The visible dots resembling colonies are overgrown areas of cells but not real colonies because they did not originate from a single cell. This figure is representative of four independent experiments.
